# Mechanical Thrombectomy in Acute Ischemic Stroke: A Systematic Review

**DOI:** 10.1017/cjn.2016.30

**Published:** 2016-04-13

**Authors:** Anna Lambrinos, Alexis K. Schaink, Irfan Dhalla, Timo Krings, Leanne K. Casaubon, Nancy Sikich, Cheemun Lum, Aditya Bharatha, Vitor Mendes Pereira, Grant Stotts, Gustavo Saposnik, Linda Kelloway, Xuanqian Xie, Michael D. Hill

**Affiliations:** 1 Evidence Development and Standards, Health Quality Ontario, Toronto, ON, Canada; 2 Departments of Medical Imaging and Surgery, University of Toronto, ON, Canada; 3 Toronto Western Hospital and University Health Network, Toronto, ON, Canada; 4 Department of Medicine, Division of Neurology, University of Toronto, Toronto, ON, Canada; 5 Interventional Neuroradiology, The Ottawa Hospital, Ottawa Hospital Research Institute, Ottawa, ON, Canada; 6 Neuroradiology Department of Medical Imaging, St. Michael’s Hospital, Toronto, ON, Canada; 7 Ottawa Stroke Program, Ottawa, ON, Canada; 8 Stroke Outcomes Research Centre, St. Michael’s Hospital, University of Toronto, Toronto, ON, Canada; 9 Ontario Stroke Network, ON, Canada; 10 Toronto Health Economics and Technology Assessment Collaborative, Leslie Dan Pharmacy, University of Toronto, Toronto, ON, Canada; 11 Department of Clinical Neurosciences, Hotchkiss Brain Institute, Cumming School of Medicine, University of Calgary, Calgary, AB, Canada

**Keywords:** acute ischemic stroke, endovascular treatment, mechanical thrombectomy, meta-analysis, systematic review

## Abstract

Although intravenous thrombolysis increases the probability of a good functional outcome in carefully selected patients with acute ischemic stroke, a substantial proportion of patients who receive thrombolysis do not have a good outcome. Several recent trials of mechanical thrombectomy appear to indicate that this treatment may be superior to thrombolysis. We therefore conducted a systematic review and meta-analysis to evaluate the clinical effectiveness and safety of new-generation mechanical thrombectomy devices with intravenous thrombolysis (if eligible) compared with intravenous thrombolysis (if eligible) in patients with acute ischemic stroke caused by a proximal intracranial occlusion. We systematically searched seven databases for randomized controlled trials published between January 2005 and March 2015 comparing stent retrievers or thromboaspiration devices with best medical therapy (with or without intravenous thrombolysis) in adults with acute ischemic stroke. We assessed risk of bias and overall quality of the included trials. We combined the data using a fixed or random effects meta-analysis, where appropriate. We identified 1579 studies; of these, we evaluated 122 full-text papers and included five randomized control trials (n=1287). Compared with patients treated medically, patients who received mechanical thrombectomy were more likely to be functionally independent as measured by a modified Rankin score of 0-2 (odds ratio, 2.39; 95% confidence interval, 1.88-3.04; I^2^=0%). This finding was robust to subgroup analysis. Mortality and symptomatic intracerebral hemorrhage were not significantly different between the two groups. Mechanical thrombectomy significantly improves functional independence in appropriately selected patients with acute ischemic stroke.

## RÉSUMÉ

**Étude systématique de l’utilisation de la thrombectomie mécanique dans les cas d’AVC ischémiques aigus.** Bien que la thrombolyse intraveineuse augmente la probabilité d’une récupération fonctionnelle satisfaisante chez des patients dûment sélectionnés ayant souffert d’un AVC ischémique aigu, une proportion élevée de patients ayant bénéficié de cette technique médicale peinent à récupérer adéquatement. Plusieurs essais cliniques récents en matière de thrombectomie mécanique semblent indiquer que ce traitement pourrait être supérieur à la thrombolyse. Nous avons ainsi procédé de façon systématique à une recension méta-analytique devant évaluer l’efficacité clinique et l’innocuité d’une nouvelle génération de dispositifs mécaniques de thrombectomie en comparaison avec la thrombolyse intraveineuse administrée, sous réserve de certains critères d’admissibilité, à des patients ayant souffert d’un AVC ischémique aigu causé par une occlusion intracrânienne proximale. À cet égard, nous avons systématiquement interrogé sept bases de données afin de retracer des essais randomisés contrôlés dont les résultats ont été publiés entre janvier 2005 et mars 2015. Ces essais comparaient l’efficacité des extracteurs d’endoprothèses (*stent retrievers*) ou des dispositifs de thrombo-aspiration aux meilleurs traitements médicaux, accompagnés ou non de la thrombolyse intraveineuse, chez des adultes ayant souffert d’un AVC ischémique aigu. Nous avons aussi évalué la qualité d’ensemble ainsi que les risques de biais de ces essais randomisés. Le cas échéant, nous avons combiné les données au moyen d’une méta-analyse à effets aléatoires ou fixes. Au total, nous avons repéré 1579 études; de ce nombre, nous en avons évalué 122 dans leur intégralité et inclus cinq essais randomisés contrôlés (n = 1287). Comparativement à des patients ayant reçu un traitement médical « conservateur », ceux ayant bénéficié d’une thrombectomie mécanique étaient plus susceptibles de récupérer leur autonomie fonctionnelle, laquelle a été mesurée par l’échelle de Rankin modifiée entre 0 et 2 (rapport des chances ou *odds ratio* : 2,39; intervalle de confiance à 95% ; 1,88-3,04; I2 = 0%). Les résultats d’analyse de ce sous-groupe se sont également révélés robustes. Tant la mortalité que des manifestations d’hémorragie intracérébrale symptomatiques ne se sont pas apparues foncièrement différentes d’un groupe à l’autre. On peut donc conclure que la thrombectomie mécanique améliore de façon notable l’autonomie fonctionnelle de patients dûment sélectionnés ayant souffert d’un AVC ischémique aigu.

## Introduction

Acute ischemic stroke is a leading cause of death and disability.^1^ ^,^ ^2^ In patients who are diagnosed promptly, acute treatment can include an attempt to reestablish blood flow. Although intravenous thrombolysis (IVT) has been routinely used to achieve this objective for approximately 20 years, this treatment has two key limitations: first, it must be administered within 4.5 hours of symptom onset; second, it has many contraindications (e.g. recent surgery, active bleeding, coagulation abnormalities).^3^ In addition, IVT is not nearly as effective in reestablishing blood flow when a thrombus is occluding a large artery such as the middle cerebral artery or the distal carotid artery, and strokes due to occlusions of these arteries are especially disabling. The key clinical outcome of interest is functional independence, and both physiological and clinical evidence to support the hypothesis that early recanalization is associated with better functional outcomes.^4^ ^-^ ^11^

Efforts to improve upon IVT, either through intra-arterial administration or thrombolytic therapy or through first-generation mechanical thrombectomy devices, were initially unsuccessful.^12^ ^-^ ^14^ However, several recent randomized trials (RCTs) of newer generation mechanical thrombectomy devices (retrievable stents: a wire mesh cage that expands to capture the clot and remove it; second-generation thromboaspiration: a combined clot extraction using aspiration and debulking of clot) have shown the potential to improve clinical and functional outcomes in patients with acute ischemic stroke resulting from occlusion of a large artery.

We evaluated the clinical effectiveness and safety of new-generation mechanical thrombectomy devices (with IVT, if eligible) compared with IVT (if eligible) in patients with acute ischemic stroke caused by a proximal intracranial occlusion in the anterior circulation (carotid artery termination, middle cerebral artery, or anterior cerebral artery). Outcomes of interest were functional independence, mortality, symptomatic intracerebral hemorrhage, recanalization, and reperfusion rates.

## Methods

### Data Sources and Study Selection

A medical librarian performed a systematic search of the articles published between January 1, 2005, and March 11, 2015, using Ovid MEDLINE, Embase, Cochrane Database of Systematic Reviews, Database of Abstracts of Reviews of Effects, CRD Health Technology Assessment Database, Cochrane Central Register of Controlled Trials, and NHS Economic Evaluation Database for relevant articles using search terms and word variants for “brain ischemia,” “stroke,” and “thrombectomy.” The full search strategy is outlined in Supplementary Appendix 1. Title and abstracts of all citations were assessed for inclusion by a single reviewer (AL) and, for those studies meeting the eligibility criteria, full-text articles were obtained. Reference lists were also examined for any additional relevant studies not identified through the search. The literature search was updated on weekly basis until June 30, 2015.

We included RCTs that enrolled patients who presented with acute ischemic stroke caused by proximal intracranial arterial occlusion of the anterior circulation (intracranial internal carotid artery, M1-middle cerebral artery [MCA] or M2-MCA branches, A1-anterior cerebral artery branches) presenting in the hospital within 12 hours of stroke onset with disabling neurological deficits. Patients were adults aged 18 years and older and were functionally independent before stroke. Methods of active intervention included mechanical thrombectomy using stent retrievers and thromboaspiration devices. We only included new mechanical thrombectomy devices given RCTs demonstrated highly significant differences between “old”-generation devices and the newer generation.^15^ ^,^ ^16^ Mechanical thrombectomy alone or as an adjunct to IVT was included. We included trials that treated the control group with IVT or best medical therapy as described in guidelines for the management of acute stroke.^17^ ^,^ ^18^ Included trials had to have at least 3 months of follow-up. Trials that use imaging-based methods to triage patients were included. We excluded trials that included patients with an occlusion of the basilar artery, those who presented in the hospital more than 12 hours after stroke onset, and trials evaluating mechanical thrombectomy with “off-label” devices. We included English-language. full-text RCTs published in peer-reviewed journals.

The primary outcomes were functional independence, mortality, and symptomatic intracerebral hemorrhage. Secondary outcomes were recanalization and reperfusion rates.

### Data Extraction and Quality Assessment

A single author (AL) extracted data and another author (AKS) verified the following data from trials: age, sex, type of occlusion, National Institutes of Health stroke scale score, Alberta stroke program early CT score, treatment protocol for intervention and control groups, status of IVT, and information to assess methodological quality. The outcomes of interest were extracted from the individual trials for both intervention and control arms: functional independence as measured by the modified Rankin scale (the modified Rankin score is a 7-point scale ranging from 0 (no symptoms) to 6 (death); a score of ≤2 indicates functional independence), mortality, symptomatic intracerebral hemorrhage, and recanalization and reperfusion rates. If data were not provided, we contacted the authors of the trial to obtain results.

The quality of the body of evidence for each outcome was examined according to the Grading of Recommendations Assessment, Development, and Evaluation Working Group criteria.^19^ The overall quality of each outcome was determined to be high, moderate, low, or very low using a step-wise, structured methodology. Two authors (AL and AKS) independently assessed the overall quality of the evidence.

### Data Synthesis

For the primary and subgroup analysis, we calculated pooled odds ratio (OR) estimates and 95% confidence intervals (CIs) using fixed-effects models, where appropriate. The degree of statistical heterogeneity among studies was assessed using the I^2^ statistic, where I^2^>50% was considered to be the cutoff point for moderate heterogeneity.^20^ A random effects model was used if the I^2^ statistic was >50%. In a subgroup meta-analysis, we investigated whether effectiveness of mechanical thrombectomy on functional independence differed according to age of patient (≤70 years or >70 years), status of IVT (IVT eligible or IVT ineligible), location of occlusion (internal carotid artery or MCA). We used Review Manager Version 5.2^21^ to conduct the meta-analyses.

## Results

**Figure 1:**
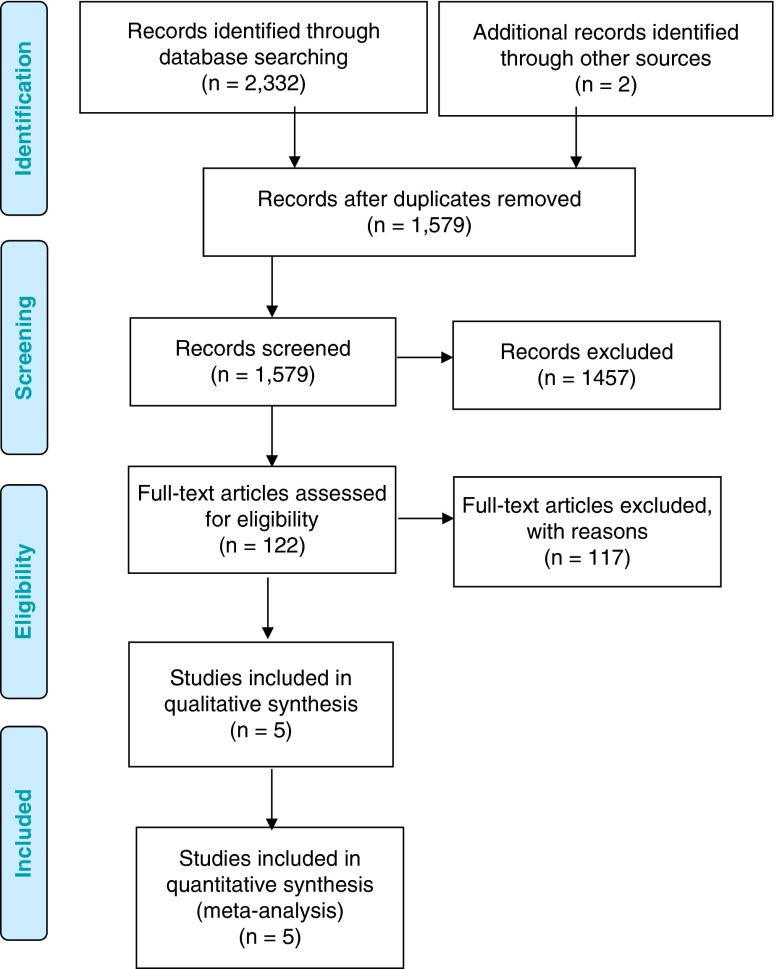
Preferred Reporting Items for systematic reviews and meta-analyses flow diagram. Adapted from Moher et al.^31^

The database search yielded 1579 relevant citations (duplicates removed) published between January 1, 2005, and March 11, 2015. The selection of trials for our analysis is summarized in Figure 1. After the screening of titles and abstracts, 122 articles were reviewed in full. We excluded 117 because of nonrandomized design (n=72), protocol or conference abstract (n=13), no comparator or IVT alone comparator (n=23), older technique/generation or “off-label” device (n=7), and irrelevance to the research question (n=2). Nonrandomized trials were included until final text screening to assist with economic modelling (results reported elsewhere). Five RCTs^22^ ^-^ ^26^ met the inclusion criteria. The reference lists of the included studies were hand-searched and no other articles were identified.

The RCTs included patients from multiple sites in 13 countries (Australia, Austria, Canada, Denmark, France, Germany, Ireland, Netherlands, New Zealand, South Korea, Spain, United Kingdom, United States). Inclusion criteria were similar across RCTs: adults aged 18 years and older, functionally independent before stroke, and most patients had an occlusion of the distal intracranial carotid artery or middle cerebral artery (M1 or M2). Randomization methodology used web-based approaches for stratified randomization^22^ ^,^ ^23^ ^,^ ^25^ or randomized minimization algorithms.^24^ ^,^ ^26^ Outcome assessment was blinded in all five trials. Baseline characteristics of intervention and control arms in the included RCTs were evenly distributed and all RCTs also had 90-day follow-up. Table 1 presents the baseline characteristics for both intervention and control study samples. Risk of bias in the five included RCTs was generally low. The assessment of risk of bias and the quality of evidence can be found in Supplementary Appendix 2.

**Table 1:** Baseline characteristics of included RCTs

Treatment group (n)	Age* Male, n (%)	Type of occlusion, n (%)	NIHSS (range)	Status of IVT, n (%)
Berkhemer et al.^22^				
MT (n=233)	65.8 (54.5-76.0)	Intracranial ICA: 1 (0.4)	17 (14-21)	IVT: 203 (87.1)
	135 (57.9)	ICA with M1 MCA: 59 (25.3)		No IVT: 30 (12.9)
		M1 MCA: 154 (66.1)		
		M2 MCA: 18 (7.7)		
		A1 or A2: 1 (0.4)		
		Extracranial ICA: 75 (32.2)		
BMT (n=267)	65.7 (55.5-76.4)	Intracranial ICA: 3 (1.1)	18 (14-22)	IVT: 242 (90.6)
		ICA with M1 MCA: 75 (28.2)		No IVT: 25 (9.4)
	157 (58.8)	M1: 165 (62.0)		
		M2: 21 (7.9)		
		A1 or A2: 2 (0.8)		
		Extracranial ICA: 70 (26.3)		
Campbell et al.^23^				
MT (n=35)	68.6 ± 12.3	ICA: 11 (31)	17 (13-20)	All patients received IVT
	17 (49)	M1 MCA: 20 (57)		
		M2 MCA: 4 (11)		
BMT (n=35)	70.2 ± 11.8	ICA: 11 (31)	13 (9-19)	All patients received IVT
	17 (49)	M1: 18 (51)		
		M2: 6 (17)		
Goyal et al.^24^				
MT (n=165)	71 (60-81)	ICA with M1 MCA: 45/163 (27.6)	16 (13-20)	IVT: 120 (72.7)
	79 (47.9)	M1 or all M2 MCA: 111/163 (68.1)		No IVT: 45 (27.3)
		Single M2 MCA: 6/163 (3.7)		
		Ipsilateral CC + I/MCA: 21 (12.7)		
BMT (n=150)	70 (60-81)	ICA with M1 MCA: 39/147 (26.5)	17 (12-20)	IVT: 118 (78.7)
	71 (47.3)	M1 or all M2 MCA: 105/147 (71.4)		No IVT: 32 (21.3)
		Single M2 MCA: 3/147 (2.0)		
		Ipsilateral CC + I/MCA: 19 (12.7)		
Jovin et al.^25^				
MT (n=103)	65.7 ± 11.3	Intracranial ICA without M1: 0 (0)	17 (14-20)	IVT: 70 (68.0)
	55 (53.4)	Terminal ICA with M1 MCA: 26/102 (25.5)		No IVT: 32 (32.0)
		M1 MCA: 66/102 (64.7)		
		Single M2 MCA: 10/102 (9.8)		
		Ipsilateral CC: 19/102 (18.6)		
BMT (n=103)	67.2 ± 9.5	Intracranial ICA without M1: 1/101 (1.0)	17 (12-19)	IVT: 80 (77.7)
	54 (52.4)	Terminal ICA with M1: 27/101 (26.7)		No IVT: 21 (22.3)
		M1 MCA: 65/101 (64.4)		
		Single M2 MCA: 8/101 (7.9)		
		Ipsilateral CC: 13/101 (12.9)		
Saver et al.^26^				
MT (n=98)	65.0 ± 12.5	ICA: 17/93(18.0)	17 (13-20)	All patients received IVT
	54/98 (55.0)	M1 MCA: 62/93 (67.0)		
		M2 MCA: 13/93 (14.0)		
BMT (n=98)	66.3 ± 11.3	ICA: 15/94(16.0)	17 (13-19)	All patients received IVT
	45/96 (47.0)	M1 MCA: 72/94 (77.0)		
		M2 MCA: 6/94 (6.0)		

BMT, best medical therapy; CC, cervical carotid; ICA, internal carotid artery; IVT, intravenous thrombolysis; MCA, middle cerebral artery; MT, mechanical thrombectomy; NIHSS, National Institutes of Health stroke scale; RCT, randomized clinical trial.

* Age is reported as mean ± standard deviation or median (interquartile range).

**Figure 2:**
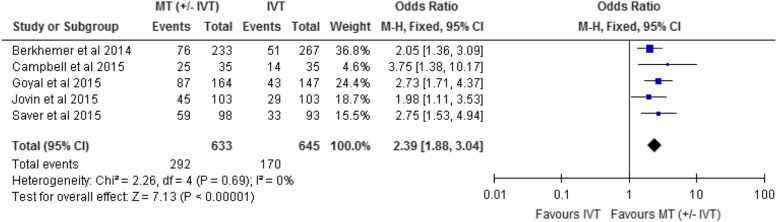
Mechanical thrombectomy versus intravenous thrombolysis on the proportion of functionally independent patients at 90-day follow-up. CI, confidence interval; IVT, intravenous thrombolysis; M-H, Mantel-Haenszel; MT, mechanical thrombectomy.

Functional independence was measured in all included RCTs at 90-day follow-up. We conducted a meta-analysis for this outcome, comparing intervention and control arms for the proportion of patients with functional independence (modified Rankin score of 0, 1, or 2). The effect of mechanical thrombectomy on functional independence was examined by pooling data from five studies with 1278 participants using a fixed-effects model (Fig. 2). There was a significant difference in functional independence between those who received mechanical thrombectomy compared with IVT (OR, 2.39; 95% CI, 1.88-3.04; I^2^=0%). We used meta-analysis to show the absolute risk difference between the two groups (Supplementary Appendix 3). The absolute risk reduction is approximately 19%; therefore, the number needed to treat is five (1/0.19). In the subgroup analyses, no significant differences in functional independence were found among subgroups of status of IVT (p=0.72), age group (p=0.24), or occlusion site (p=0.94). The subgroup analyses are shown in Supplementary Appendix 3.

**Figure 3:**
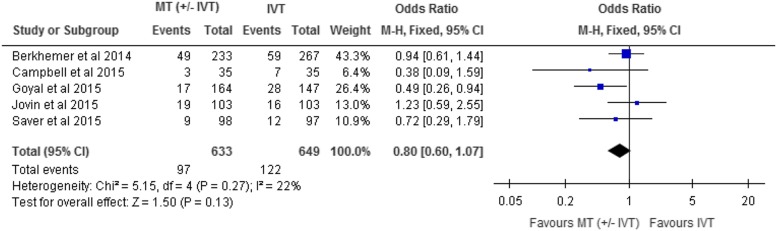
Mechanical Thrombectomy versus intravenous thrombolysis on mortality at 90-day follow-up. CI, confidence interval; IVT, intravenous thrombolysis; M-H, Mantel-Haenszel; MT, mechanical thrombectomy.

Mortality was reported in all included RCTs at 90-day follow-up. The effect of mechanical thrombectomy on mortality was examined by pooling data from five studies with 1282 participants using a fixed-effects model (Fig. 3). The difference in mortality between those who received mechanical thrombectomy and best medical therapy was not statistically significant (OR, 0.80; 95% CI, 0.60-1.07; I^2^=22%).

**Figure 4:**
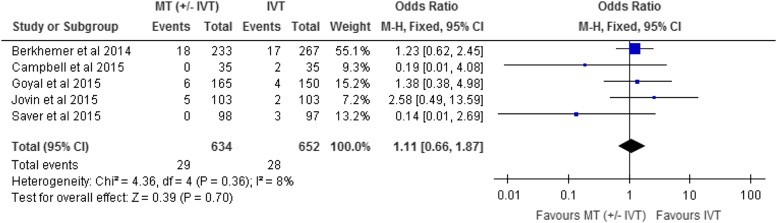
Mechanical thrombectomy versus intravenous thrombolysis on SICH at 90-day follow-up. CI, confidence interval; IVT, intravenous thrombolysis; M-H, Mantel-Haenszel; MT, mechanical thrombectomy.

Symptomatic intracerebral hemorrhage was reported as an adverse event in all included RCTs. Symptomatic intracerebral hemorrhage was clinically determined at the study sites using European-Australasian Acute Stroke Study^26^ criteria: deterioration in National Institutes of Health Stroke Scale ≥4 plus any intracerebral hemorrhage or other criteria similar to that measure.^22^ ^-25^ The effect of mechanical thrombectomy on symptomatic intracerebral hemorrhage was examined by pooling data from five studies with 1286 participants using a fixed-effects model (Fig. 4). The difference in symptomatic intracerebral hemorrhage between those who received mechanical thrombectomy and best medical therapy was not statistically significant (OR, 1.11; 95% CI, 0.66-1.87; I^2^=8%).

We did not perform meta-analyses for reperfusion and recanalization rates because of the heterogeneity of measures in the studies, but rates were higher in the intervention groups in all trials. The results for reperfusion and recanalization are presented in Supplementary Appendix 4.

## Discussion

In a systematic review of randomized trials, we found that patients with acute ischemic stroke caused by an occlusion in a large artery were considerably more likely to be functionally independent when treated with mechanical thrombectomy (with or without IVT) rather than IVT alone. The quality of evidence was rated high according to the Grading of Recommendations Assessment, Development, and Evaluation criteria.

Although we did not find a statistically significant difference in mortality between the two groups, it is worth noting that one of the included trials, there was a significant reduction in the mechanical thrombectomy group compared with best medical therapy.^24^ This study had more restrictive inclusion criteria than the other RCTs, (e.g. patients must have had only a small infarct core on computed tomography and moderate-to-good collateral circulation distal to the occlusion), and patients enrolled in this trial were treated more quickly than in the others (median time of stroke onset to reperfusion of 241 minutes compared with 324 minutes in the other trials).^12^ It is possible therefore that mechanical thrombectomy has the potential to improve not only functional independence but also survival.

The authors of two recent systematic reviews^27^ ^,^ ^28^ comparing endovascular treatment with IVT drew similar conclusions. The authors of both reviews included all generations of mechanical thrombectomy devices in their primary analysis and the more recently published trials in a sensitivity analysis. Our review focused on the most recent RCTs because the older mechanical thrombectomy devices are no longer on the market or in use in Canada. Patients enrolled in the older trials were also different than patients enrolled in the newer trials (e.g. many patients had no confirmation of proximal artery occlusion on imaging). It is not known if this intervention was administered optimally in their heterogenous study populations.^29^ The focus in the present review on patients appropriately triaged through imaging is more reflective of the potential application of mechanical thrombectomy in Canadian stroke practice.

Several strengths and limitations of our study merit emphasis. An advantage of our methodology is that the evidence is reviewed by methodologists who have neither intellectual nor financial conflicts of interest. This reduces the risk of bias in the selection and assessment of studies.^30^ ^,^ ^31^ Our process also includes engaging various content experts and stakeholders to receive feedback regarding any potentially missed articles. A limitation of our study is that, because of limited resources, a single author screened articles.

## Conclusion

Our systematic review and meta-analysis demonstrates that newer mechanical thrombectomy performed at specialized centres improves functional independence for patients with acute stroke compared with best medical therapy. Offering this effective treatment to all eligible patients will require careful planning and capacity building as well as the development of protocols to ensure that eligible patients are rapidly assessed in centres where mechanical thrombectomy can be offered.

## Disclosures

AB is a research fellow in an imaging department (unrelated to the manuscript) and has an unrestricted educational grant to support MS (multiple sclerosis) from Novartis. VMP is a principal investigator for the SWIFT-DIRECT trial and has received consulting fees from Medtronic. CL is a grant recipient (Ottawa Hospital Academic Medical Organization grant) from Ottawa Hospital and is a co-principal investigator under a Department of Medicine grant from Ottawa Hospital. MH is an independent investigator for Medtronic (Covidien) under a grant to the University of Calgary. LC is an independent contractor (patient assessor for SURTAVI) for and has received consulting fees from Medtronic and is a site principal investigator (no personal compensation received) for NoNO Inc. The other others have no disclosures.

The views and opinions expressed by the authors in this publication are those of the authors and do not necessarily reflect those of Health Quality Ontario.

## Supplementary material

For supplementary material accompanying this paper visit http://dx.doi.org/10.1017/cjn.2016.30. click here to view supplementary material
