# Supplementary material for: Mechanical Thrombectomy in Acute Ischemic Stroke: A Systematic Review
Source: Can J Neurol Sci. 2016 Apr 13;43(4):455–60. doi: 10.1017/cjn.2016.30 (PMC4926268; doi:10.1017/cjn.2016.30)
Supplement: Supplementary file 1 [file S0317167116000305sup001.docx]

**APPENDICES**

**Appendix 1: Literature Search Strategy**

**Appendix 2: Evidence Quality Assessment**

**Appendix 3: Functional Independence Risk Difference and Subgroup Results**

**Appendix 4: Secondary Outcome Results**

# Appendices

## Appendix 1: Literature Search Strategies

Database: EBM Reviews - Cochrane Central Register of Controlled Trials <February 2015>, EBM Reviews - Cochrane Database of Systematic Reviews <2005 to January 2015>, EBM Reviews - Database of Abstracts of Reviews of Effects <1st Quarter 2015>, EBM Reviews - Health Technology Assessment <1st Quarter 2015>, EBM Reviews - NHS Economic Evaluation Database <1st Quarter 2015>, Embase <1980 to 2015 Week 10>, All Ovid MEDLINE(R) <1946 to Present>

Search Strategy:

--------------------------------------------------------------------------------

1 exp Brain Ischemia/ (198337)

2 ((isch?emi* adj3 (stroke* or apoplex* or cerebr* or brain or encephalopath* or neur*)) or AIS).tw. (198076)

3 exp Stroke/ (189703)

4 (stroke* adj3 (acute or cerebr* or attack* or accident* or lacunar or cardioembol*)).tw. (76907)

5 Intracranial Arteriosclerosis/ (10347)

6 exp "Intracranial Embolism and Thrombosis"/ (367169)

7 Carotid Artery Thrombosis/ (5356)

8 ((occlus* or block* or infarct* or clot* or termination) adj6 (carotid or cerebr* or MCA or ACA)).tw. (96218)

9 or/1-8 (786703)

10 exp Thrombectomy/ (15008)

11 Embolectomy/ (4255)

12 ((Mechanical adj3 (thromb* or embol* or clot disruption* or clot retrieval*)) or ((clot* or thromb* or embol*) adj3 (retriev* or disruption* or fragmentation)) or ((stent* or stent-assisted) adj3 retriev*) or stentriever*).tw. (9056)

13 ((Merci or Trevo or Penumbra or Solitaire) adj3 (retriever* or system* or device*)).mp. (1233)

14 or/10-13 (24914)

15 9 and 14 (14515)

16 exp Animals/ not (exp Animals/ and Humans/) (8045601)

17 15 not 16 (14213)

18 (case reports or congresses).pt. (1780093)

19 17 not 18 (13866)

20 limit 19 to english language [Limit not valid in CDSR,DARE; records were retained] (12321)

21 limit 20 to yr="2005 -Current" [Limit not valid in DARE; records were retained] (9737)

22 21 use pmoz,cctr,coch,dare,clhta,cleed (970)

23 exp Brain Ischemia/ (198337)

24 ((isch?emi* adj3 (stroke* or apoplex* or cerebr* or brain or encephalopath* or neur*)) or AIS).tw. (198076)

25 exp Cerebrovascular Accident/ (189703)

26 Stroke Patient/ (13478)

27 (stroke* adj3 (acute or cerebr* or attack* or accident* or lacunar or cardioembolic)).tw. (76838)

28 exp Occlusive Cerebrovascular Disease/ (26483)

29 exp Carotid Artery Obstruction/ (25862)

30 Brain Embolism/ (8515)

31 ((occlus* or block* or infarct* or clot* or termination) adj6 (carotid or cerebr* or MCA or ACA)).tw. (96218)

32 or/23-31 (478286)

33 Mechanical Thrombectomy/ (1828)

34 Thrombectomy/ (10732)

35 Embolectomy/ (4255)

36 ((Mechanical adj3 (thromb* or embol* or clot disruption* or clot retrieval*)) or ((clot* or thromb* or embol*) adj3 (retriev* or disruption* or fragmentation)) or ((stent* or stent-assisted) adj3 retriev*) or stentriever*).tw. (9056)

37 ((Merci or Trevo or Penumbra or Solitaire) adj3 (retriever* or system* or device*)).mp. (1233)

38 or/33-37 (22583)

39 32 and 38 (4742)

40 exp animal experimentation/ or exp models animal/ or exp animal experiment/ or nonhuman/ or exp vertebrate/ (38090949)

41 exp humans/ or exp human experimentation/ or exp human experiment/ (29700691)

42 40 not 41 (8416467)

43 39 not 42 (4642)

44 case report/ or conference abstract.pt. (5381946)

45 43 not 44 (2728)

46 limit 45 to english language [Limit not valid in CDSR,DARE; records were retained] (2466)

47 limit 46 to yr="2005 -Current" [Limit not valid in DARE; records were retained] (2248)

48 47 use emez (1362)

49 22 or 48 (2332)

50 remove duplicates from 49 (1624)

## Appendix 2: Evidence Quality Assessment

Table A1: GRADE Evidence Profile for Comparison of Mechanical Thrombectomy and Best Medical Therapy on Clinical Outcomes

| **Number of Studies (Design)** | **Risk of Bias** | **Inconsistency** | **Indirectness** | **Imprecision** | **Publication Bias** | **Upgrade Considerations** | **Quality** |
| --- | --- | --- | --- | --- | --- | --- | --- |
| **Functional Independence (mRS)** | | | | | | | |
| 5 (RCTs) | No serious limitations | No serious limitations | No serious limitations | No serious limitations^a^ | Undetected | No other considerations | ⊕⊕⊕⊕ High |
| **Mortality** |  |  |  |  |  |  |  |
| 5 (RCTs) | No serious limitations | No serious limitations | No serious limitations | Serious limitations (–1)^a^ | Undetected | No other considerations | ⊕⊕⊕ Moderate |
| **SICH** |  |  |  |  |  |  |  |
| 5 (RCTs) | No serious limitations | No serious limitations | No serious limitations | Serious limitations (–1)^a^ | Undetected | No other considerations | ⊕⊕⊕ Moderate |
| **Reperfusion** |  |  |  |  |  |  |  |
| 5 (RCTs) | No serious limitations | No serious limitations | No serious limitations^b^ | Serious limitations (–1)^a^ | Undetected | No other considerations | ⊕⊕⊕ Moderate |
| **Recanalization** |  |  |  |  |  |  |  |
| 3 (RCTs) | No serious limitations | No serious limitations | Serious limitations (–1)^c^ | Serious limitations (–1)^a^ | Undetected | No other considerations | ⊕⊕ Low |

Abbreviations: mRS, modified rankin scale; SICH, symptomatic intracranial hemorrhage

^a^Optimal information size (OIS) may not be met for this outcome as 4 out of 5 RCTs were stopped early.

^b^Reperfusion can be considered a surrogate outcome.

^c^Recanalization can be considered a surrogate outcome.

Table A2: Risk of Bias Among Randomized Controlled Trials for the Comparison of Mechanical Thrombectomy and Clinical Outcomes

| **Author, Year** | **Allocation Concealment^a^** | **Blinding^b^** | **Complete Accounting of Patients and Outcome Events** | **Selective Reporting Bias** | **Other Limitations** |
| --- | --- | --- | --- | --- | --- |
| Berkhemer et al, 2014 | No limitations | No limitations | No limitations | Limitations^c^ | No limitations^f^ |
| Campbell et al, 2015 | No limitations | No limitations | No limitations | No limitations | Limitations^e,f^ |
| Goyal et al, 2015 | No limitations | No limitations | No limitations | Limitations^d^ | Limitations^e,f^ |
| Jovin et al, 2015 | No limitations | No limitations | No limitations | No limitations | Limitations^e,f^ |
| Saver et al, 2015 | No limitations | No limitations | No limitations | No limitations | Limitations^e,f^ |

Abbreviations:

^a^All 5 RCTs used a web-based randomized minimization procedure.

^b^All 5 included RCTs had blind outcome evaluation but physicians conducting the intervention were aware of treatment assignment. This was appropriate as a sham procedure was not ethical and standard of care is intravenous thrombolysis which is a more appropriate comparator.

^c^RCT protocol states the functional outcome measured by ‘Academic Linear Disability Scale’ would be collected at 90 days, but this outcome is not reported in the published article.

^d^RCT protocol states the functional outcome measured by ‘miFUNCTION scale’ would be collected, but this outcome is not reported in the published article.

^e^After the Berkhemer et al (2015) study was presented at the World Stroke Conference in October 2014, the 4 following RCTs stopped early based on prespecified boundary for efficacy during interim analysis.

^f^All studies had grant support from Covidien/ev3 (the company that manufactures the Solitaire FR stent retriever) and/or other manufacturers/industry support.

## Appendix 3: Subgroup Results


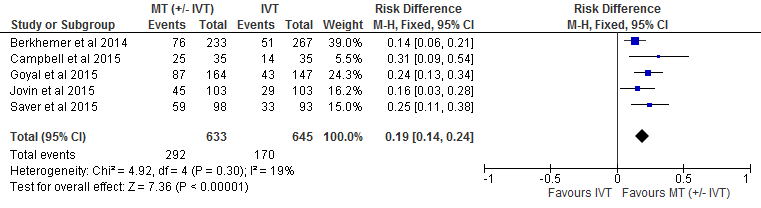


Figure A1: Risk Difference of Mechanical Thrombectomy Versus Best Medical Therapy on Proportion of Functionally Independent Patients at 90-Day Follow-up


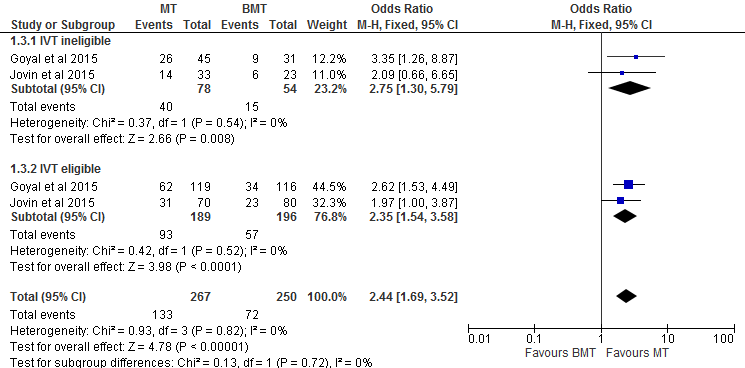


Figure A2: Mechanical Thrombectomy Versus Best Medical Therapy on the Proportion of Functionally Independent Patients at 90-Day Follow-up by Status of IVT


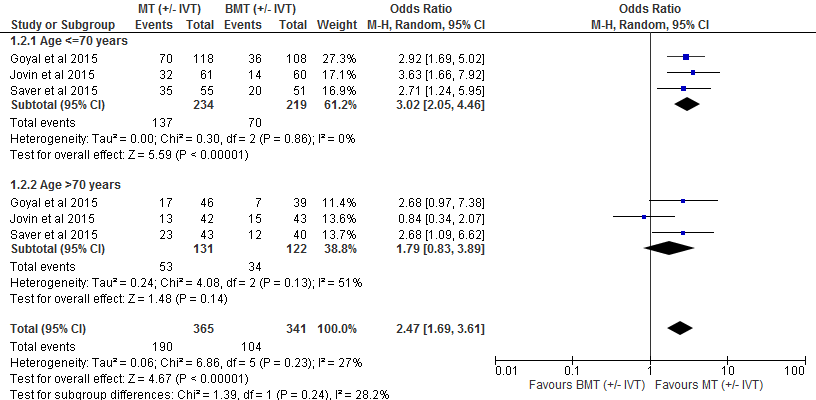


*****Goyal et al divided age in <=80 years and >80 years in the age sensitivity analysis.

Figure A3: Mechanical Thrombectomy Versus Best Medical Therapy on the Proportion of Functionally Independent Patients at 90-Day Follow-up by Age, Secondary Analysis

**
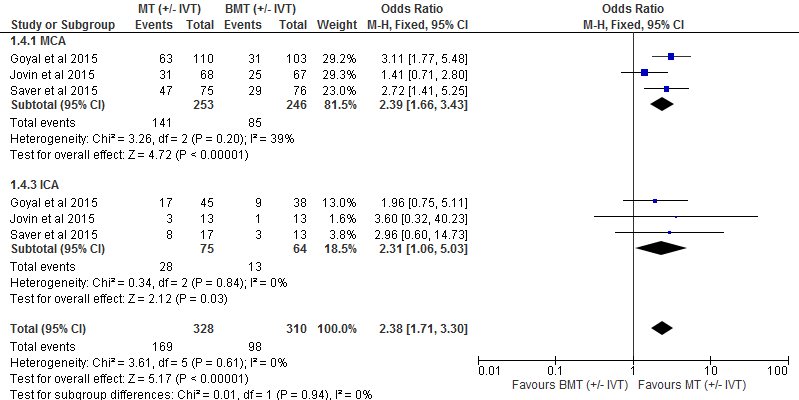
**

Figure A4: Mechanical Thrombectomy Versus Best Medical Therapy on the Proportion of Functionally Independent Patients at 90-Day Follow-up by Occlusion Site


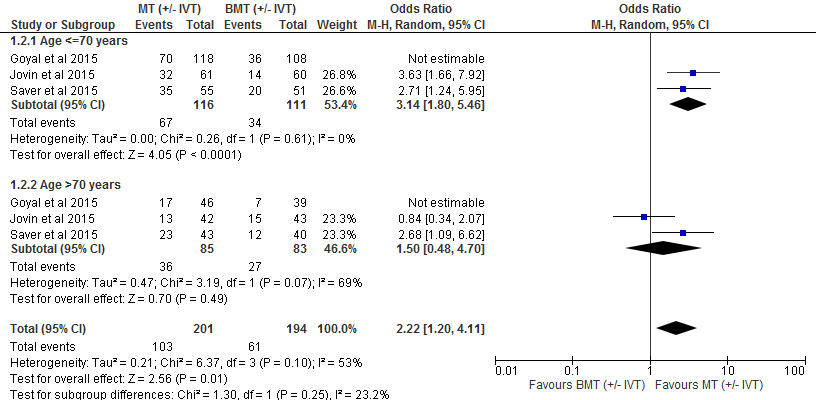


*Goyal et al estimate not included in this sensitivity analysis as this trial divided patients into <=80 years and >80 years and we wanted to determine if the estimate influenced results.

Figure A5: Mechanical Thrombectomy Versus Best Medical Therapy on Proportion of Functionally Independent Patients at 90-Day Follow-up by Age, Secondary Analysis without ESCAPE Study

## Appendix 4: Secondary Outcome Results

Table 1: Reperfusion and Recanalization Rates in Included RCTs

| **Author, Year** | **Intervention** | **Control** | **Adjusted Value (95% CI)** |
| --- | --- | --- | --- |
| **Berkhemer et al** |  |  |  |
| Reperfusion | 115/196 (58.7%)^a^ | NR | NR |
| Recanalization | 141/187 (75.4%)^b^ | 68/207 (32.9%)^b^ | 6.88 (4.34 to 10.94) |
| **Campbell et al** |  |  |  |
| Reperfusion | 100 (100 to 100)^c^ | 37 (-0.5 to 96) | 4.7 (2.5 to 9.0)^d^ |
| Recanalization | 33 (94%)^e^ | 15 (43%)^e^ | 29.0 (5.4 to 155.0)^d^ |
| **Goyal et al** |  |  |  |
| Reperfusion | 113/156 (72.4%)^f^:  79/112 (70.5%) with IVT  34/44 (77%) w/o IVT | NR | NR |
| Recanalization | NR | 43/138 (31.2%)^g^:  41/110 (32.3%) with IVT  2/28 (7%) w/o IVT | NR |
| **Jovin et al** |  |  |  |
| Reperfusion | 67/102 (65.7%)^a^ | NR | NR |
| Recanalization | NR | NR | NR |
| **Saver et al** |  |  |  |
| Reperfusion | 73/83 (88%)^a^  53/64 (83%)^h^ | 21/52 (40%) | 2.05 (1.45 to 2.91)^d^ |
| Recanalization | NR | NR | NR |

Abbreviations: IVT, intravenous thrombolysis, NR, not reported

^a^Reperfusion was measured by the modified Thrombolysis in Cerebral Infarction (TICI) score where a score of 2b or 3, indicating complete filling of the expected vascular territory – no./total no. (%).

^b^Measured as number of patients with no intracranial occlusion on follow-up CT angiography – no./total no. (%).Data for follow-up CT angiography were not available for 106 patients owing to imminent death or death (24 patients), decreased kidney function (13 patients), insufficient scan quality (5 patients), and other reasons (64 patients).

^c^Reperfusion was defined as the percentage reduction in the perfusion-lesion volume between initial imaging and 24-hour imaging (IQR). This value can be negative if hypoperfusion becomes more severe over time. This analysis was adjusted for the site of vessel occlusion at baseline.

^d^P<0.001.

^e^Recanalization was defined as a Thrombolysis in Myocardial Infarction score of 2 or 3 (partial or complete restoration of flow at the site of arterial occlusion) and measured at 24 hours. This analysis was adjusted for the site of vessel occlusion at baseline.

^f^Reperfusion was measured by the Thrombolysis in Cerebral Infarction (TICI) score where a score of 2b or 3, indicating complete filling of the expected vascular territory – no./total no. (%).

^g^Recanalization was measured by a modified Arterial Occlusive Lesion (AOL) score of 2 or 3 indicates partial or complete recanalization – no./total no.

^h^Saver et al also reported successful reperfusion at 27 hr. – no./total no (%). Successful reperfusion was defined as reperfusion at least 90%, as assessed with the use of perfusion CT or MRI. Data on successful reperfusion were not obtained for all patients after the adoption of the protocol amendment making penumbral imaging optional
